# Repeatability, reproducibility and agreement of foveal avascular zone measurements using three different optical coherence tomography angiography devices

**DOI:** 10.1371/journal.pone.0206045

**Published:** 2018-10-18

**Authors:** Natasa Mihailovic, Cristin Brand, Larissa Lahme, Friederike Schubert, Eike Bormann, Nicole Eter, Maged Alnawaiseh

**Affiliations:** 1 Department of Ophthalmology, University of Muenster Medical Center, Muenster, Germany; 2 Institute of Reproductive and Regenerative Biology, Center of Reproductive Medicine and Andrology, University of Muenster, Muenster, Germany; 3 Institute of Biostatistics and Clinical Research, University of Muenster, Muenster, Germany, Muenster, Germany; Save Sight Institute, AUSTRALIA

## Abstract

**Purpose:**

To evaluate the repeatability, the reproducibility and the agreement of foveal avascular zone (FAZ) measurements using three different optical coherence tomography angiography (OCT-A) devices.

**Procedures:**

This prospective study included 24 eyes of 24 healthy volunteers. OCT-A imaging was performed using RTVue XR Avanti, Canon OCT-HS100 and Spectralis OCT-A. Repeated measurements were performed under the same conditions on two separate days, and the area of the FAZ was determined and analyzed using the above devices.

**Results:**

All three devices showed a high ICC and there was no significant difference between the ICCs (pairwise comparison) of the three devices (Optovue–Canon (p = 0.66); Canon–Heidelberg (p = 0.21); Heidelberg–Optovue (p = 0.37). Agreement analysis of the three devices revealed a significant elevation of FAZ area values with the Heidelberg device and a slight underestimation of the FAZ area with the Canon device. Nevertheless, overall we found a high level of agreement between all of the three devices (ICC ≥ 0.958 (0.905–0.982)).

**Conclusions:**

Good reproducibility and repeatability were observed for all three devices. However, the agreement analysis revealed slight, but significant differences, which might limit alternating use of these devices for clinical research and follow-up examinations.

## Introduction

Optical coherence tomography angiography (OCT-A) is an innovative technology, enabling the visualization of the chorioretinal vasculature [1]. Unlike fluorescein or indocyanine green angiography (ICG), OCT-A has the advantage of being non-invasive and not requiring application of intravenous dye, since its technology uses the movement of the red blood cells to calculate flow [2]. Consequently, this also means that phenomena such as pooling or staining do not occur in OCT-A, since there is no dye leaking from pathological microvasculature. This allows better visualization of the whole microvasculature including the foveal avascular zone (FAZ) in healthy subjects and also facilitates investigation of retinal pathologies such as diabetic retinopathy and the examination of eyes with retinal neovascularisations [3,4,5].

Analysis of FAZ parameters and the correlation of OCT-A measurements with visual acuity and disease severity has attracted increasing interest over the last few years and has been described in patients with different retinal diseases such as diabetic retinopathy, retinal vein occlusion and geographic atrophy [6,7,8]. OCT-A is a rapidly developing imaging technology and a number of different OCT-A instruments, developed by different companies, are currently used in clinical practice.

There are different studies in the literature, which evaluated measurements of the FAZ area using different OCT-A devices [9,10]. Some of these studies used an external software program to measure the FAZ area. In particular, this is usually not applicable in the clinical practice. The aim of this study was to evaluate the reproducibility, repeatability and the agreement of FAZ measurements obtained in healthy subjects using following three different OCT-A devices: RTVue XR Avanti (Optovue), Canon OCT-HS100 (Canon) and Spectralis OCT-A (Heidelberg Engineering). Especially for these three devices there seems to be a lack of critical agreement analysis in the literature and it is not clear how to handle the FAZ measurements provided by these devices in clinical and scientific practice.

## Methods

A total of 25 eyes from 25 healthy volunteers with no history of any ocular and systemic disease or previous ocular surgery were examined in this prospective study. However, only 24 eyes were included, since in one case there was absence of FAZ.

The study was approved by the Ethics Committee of the University of Muenster, North Rhine Westphalia, Germany. The study adhered to the tenets of the Declaration of Helsinki and each participant signed an informed consent form prior to the OCT-A measurements.

All subjects underwent slit-lamp biomicroscopy and funduscopy, and subjects with pathological retinal findings or relevant medium opacity were excluded.

**Fig 1:**
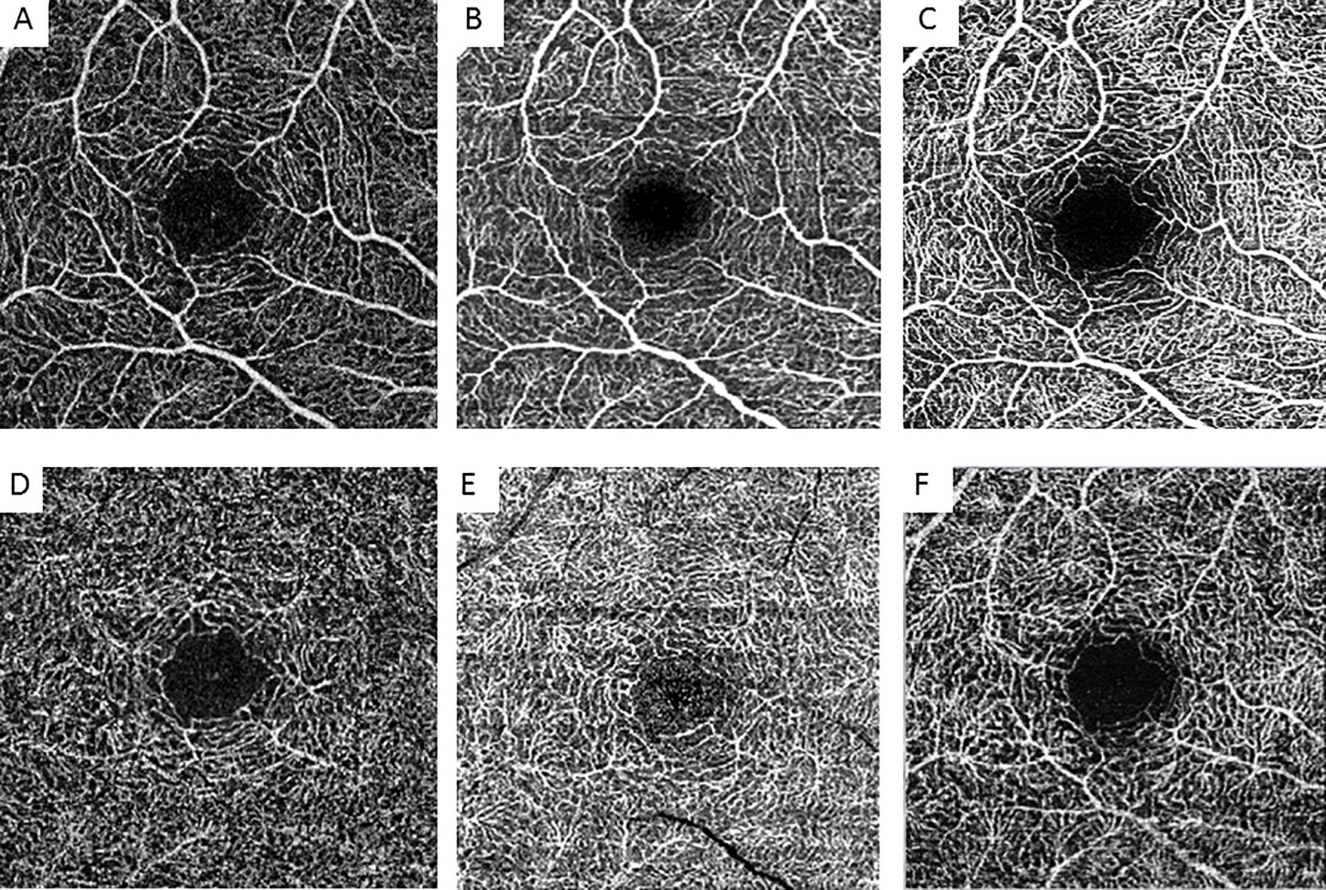
**A–F. OCT-A images.** Representative OCT-A images of the different devices of the SCP (A–C) and DCP (D–F). A+D = Optovue, B+E = Canon, C+F = Heidelberg.

In all study subjects, OCT-A images were taken by an expert examiner and performed twice with each OCT-A device on the first day of examination and once on the second. When performing imaging twice on the first day, the subjects were not moved away from the device and were only readjusted before reimaging. OCT-A imaging was performed in the right eye, using the different OCT-A devices in a randomized order. OCT-A scans were performed using a 3x3 mm macula scan (XR Avanti Angiovue, Canon OCT-HS100) or the equivalent dimension 10°x10° (Spectralis OCT-A) (Fig 1).

After imaging and checking for proper segmentation, FAZ measurement was performed in both, the superficial (SCP) and deep (DCP) OCT angiogram, for the first two measurements obtained on the first day of examination (m1, m2) and for the third measurement obtained on another day (m3) by two independent readers (R1, R2). The FAZ contours were then manually traced by the readers and the area of the FAZ was automatically calculated with the respective software provided by the manufacturers of each device.

### RTVue XR Avanti (Optovue)

This system has an A-scan rate of 70,000 scans per second, using a light source centered on 840 nm and a bandwidth of 45 nm. Each OCT-A volume contained 304 × 304 A-scans with two consecutive B-scans that were captured at each fixed position before proceeding to the next sampling location. Image quality was optimized using the “autocorrect” function. The maximum signal strength index (SSI) is 100. Any images with a SSI <60 were excluded from the study. The SCP en face OCT-A image was segmented with an inner boundary 3 μm below the internal limiting membrane (ILM) and an outer boundary set at 15 μm below the inner plexiform layer (IPL). The DCP en face OCT-A image was segmented with an inner boundary 15 μm below the IPL and an outer boundary at 70 μm below the IPL [11].

### Canon OCT-HS100 (Canon)

The A-scan rate in this device is 70,000 scans per second, while the scanning width is between 2 and 10 mm and the wavelength used is 855 nm. The axial resolution claimed is 3 μm with a scanning depth of 2 mm [12]. The OCT angiogram area selected for this study was 3 x 3 mm. An “autocorrect” function was used before imaging to optimize the signal strength. Signal strength was portrayed on a scale of 1 to 10 and images with a signal strength <6 were excluded from the study. The SCP en face OCT angiogram was segmented with an inner boundary set at the ILM and an outer boundary set at 50 μm below the IPL, the DCP en face OCT angiogram was segmented with an inner boundary set at 50μm below the IPL and an outer boundary at the outer plexiform layer (OPL).

### Spectralis OCT-A (Heidelberg Engineering)

This device uses a wavelength of 870nm and an A-scan rate of 85kHz with an axial resolution of 7μm and a lateral resolution of 14μm [13]. An angiogram area of 10x10° was chosen as an equivalent dimension for comparison with the 3x3 mm area. In the Spectralis device, a signal strength index was not provided for the complete OCT angiogram. For quality control of the measurements provided by the device we used the quality index (QI), which needed to be at least 30 dB for the image to be included in our study (maximal QI = 50 dB). The SCP en face OCT-A image was segmented with an inner boundary set at the ILM and an outer boundary set at the outer border of the IPL. The DCP en face OCT-A image was segmented with an inner boundary set at the outer border of the IPL and an outer boundary set at the outer border of the OPL.

### Statistical analysis

Microsoft Excel 2010 was used for data management. Statistical analyses were performed using IBM SPSS Statistics 24 for Windows (IBM Corporation, Somers, NY, USA). The data was tested for normality distribution using the Kolmogorov–Smirnov test and found to fit a normal distribution. Data are generally presented as mean ± standard deviation. In order to assess the repeatability, the intraclass correlation coefficient (ICC) was calculated. Bland Altman plots were used to show the agreement between the measurements obtained using the three devices. To assess the linear relationship between measurements obtained with the Optovue device and the Canon / Heidelberg device, univariate linear regression was performed. A two-sided paired t-test was used to compare the ICC for measurements with the different devices. Inferential statistics are intended to be exploratory (i.e. forming a basis for hypotheses), rather than confirmatory, and are interpreted accordingly. The comparison-wise type-I error rate is controlled instead of the experiment-wise error rate. The global statistical significance level was set at 0.05.

## Results

24 eyes of 24 healthy subjects (age = 30.58 ± 12.11 years, 6 male, 18 female) were included. The mean FAZ area for the Optovue device was 0.312 ± 0.090 at the SCP and 0.337 ± 0.092 at the DCP, for the Canon device 0.298 ± 0.090 at the SCP and 0.302 ± 0.092 at the DCP, and for the Heidelberg device 0.329 ± 0.095 at the SCP and 0.335 ± 0.093 at the DCP, respectively. The means of the three different measurements (m1, m2, m3) as evaluated by the two readers (R1, R2) in the three different devices are shown in S1 Table.

Intraobserver repeatability for the SCP and DCP was excellent for all three devices (R1 SCP: ICC ≥ 0.970, R2 SCP: ICC ≥ 0.964; R1 DCP: ICC ≥ 0.922, R2 DCP: ICC ≥ 0.815). The interobserver intrasession reproducibility of all three measurements was also excellent for all three devices (SCP: ICC ≥ 0.967; DCP: ICC ≥ 0.921) and is shown in Table 1.

**Table 1:** Interobserver (R1 vs. R2) intrasession reproducibility of foveal avascular zone area measurements (m1, m2, m3) of the superficial and deep OCT angiogram for the three different devices.

	SCP	DCP
**Optovue**	ICC (95% CI)	ICC (95% CI)
m1	0.994 (0.987–0.998)	0.987 (0.969–0.994)
m2	0.996 (0.990–0.998)	0.956 (0.898–0.981)
m3	0.995 (0.988–0.998)	0.975 (0.943–0.989)
**Canon**		
m1	0.982 (0.959–0.992)	0.963 (0.917–0.984)
m2	0.975 (0.942–0.989)	0.951 (0.891–0.979)
m3	0.992 (0.982–0.997)	0.921 (0.826–0.965)
**Heidelberg**		
m1	0.967 (0.926–0.986)	0.975 (0.942–0.989)
m2	0.992 (0.982–0.997)	0.978 (0.951–0.991)
m3	0.986 (0.968–0.994)	0.987 (0.969–0.994)

For the same reader and the same device, no significant differences were found between m1 and m2 (Optovue: p = 0.9, Canon: p = 0.83, Heidelberg: p = 0.67) or between m1 and m3 (Optovue: p = 0.73, Canon: p = 0.70, Heidelberg: p = 0.26) (Table 2).

**Table 2:** Pairwise comparison as paired t-test between the first and second (m1 vs. m2) and the first and third measurement (m1 vs. m3) and ICC values for the three different devices (R1, SCP).

	m1: FAZ/mm^2^ (Mean±SD)	m2: FAZ/mm^2^ (Mean±SD)	m3: FAZ/mm^2^ (Mean±SD)	p-value	ICC (95% CI)
**Optovue**					
R1 (m1 vs. m2)	0.309 ± 0.087	0.301 ± 0.090	0.308 ± 0.089	0.9	0.996 (0.991–0.998)
R1 (m1 vs. m3)				0.73	0.995 (0.988–0.998)
**Canon**					
R1 (m1 vs. m2)	0.295 ± 0.093	0.295 ± 0.088	0.296 ± 0.089	0.83	0.989 (0.975–0.995)
R1 (m1 vs. m3)				0.70	0.987 (0.969–0.994)
**Heidelberg**					
R1 (m1 vs. m2)	0.326 ± 0.089	0.328 ± 0.090	0.332 ± 0.097	0.67	0.994 (0.985–0.997)
R1 (m1 vs. m3)				0.26	0.982 (0.960–0.992)

Table 3 shows the agreement between FAZ areas determined with the three devices. Although the differences were minimal, the values of the mean area of the FAZ differed significantly between all instrument pairs (Optovue–Canon: p = 0.001; Optovue–Heidelberg: p = 0.000; Canon–Heidelberg: p = 0.002).

**Table 3:** Pairwise differences and intraclass correlation coefficient (ICC) of FAZ measurements comparing the three different devices (R1, SCP, MD = mean difference, AD = absolute difference).

Device	FAZ/mm^2^	Compared to	MD	AD	p-value	ICC
**Optovue**	0.309 ± 0.087	**Canon**	0.01 ± 0.02	0.02 ± 0.01	**0.001**	0.98 (0.953–0.991)
		**Heidelberg**	-0.02 ± 0.02	0.02 ± 0.02	**0.000**	0.964 (0.919–0.984)
**Canon**	0.295 ± 0.093	**Heidelberg**	-0.03 ± 0.03	0.04 ± 0.02	**0.002**	0.958 (0.905–0.982)
**Heidelberg**	0.326 ± 0.089	-	-	-	-	-

**Fig 2:**
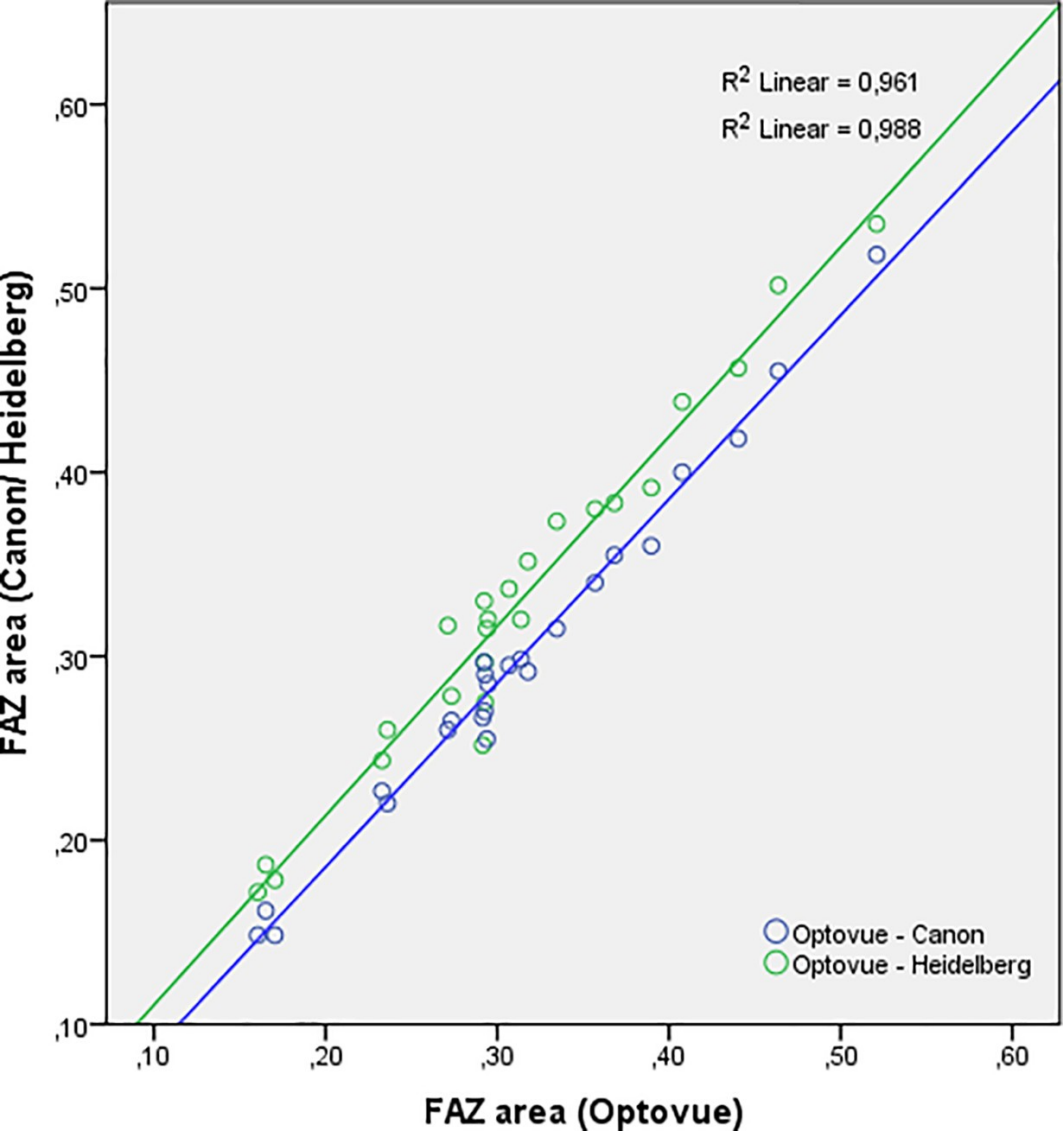
Regression analysis. Regression line showing the linear relationship between the area of the FAZ measured using the Optovue device and the Canon (blue) / Heidelberg (green) devices.

The linear relationship between measurements obtained with the Optovue device and the Canon / Heidelberg devices is shown in Fig 2. The largest area of the FAZ was measured using the Heidelberg device and the smallest area using the Canon device (Table 3 and Fig 2).

**Fig 3:**
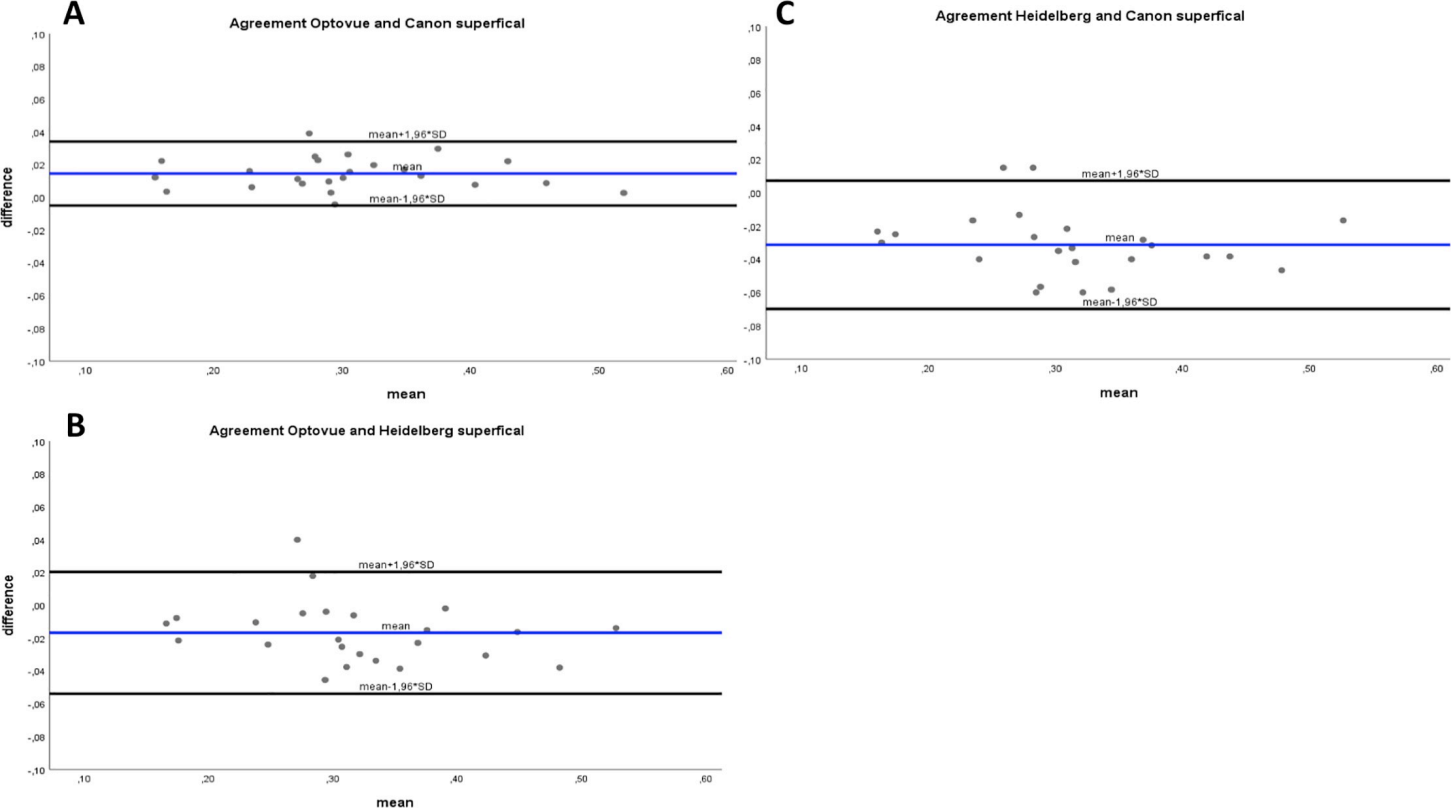
**A–C. Bland-Altman-analysis.** Bland-Altman plots showing the level of agreement for the area of the FAZ in the superficial retinal OCT-A angiogram. A: Optovue and Canon; B: Optovue and Heidelberg; C: Heidelberg and Canon. Broken line represents the mean difference; dotted lines represent the limits of agreement.

Fig 3 shows the Bland–Altman plots for the agreement between the FAZ area as measured using the three different devices. We did not find any significant difference between the ICC (pairwise comparison) of the three devices (Optovue–Canon: p = 0.66; Canon–Heidelberg: p = 0.21; Heidelberg–Optovue: p = 0.37).

## Discussion

OCT-A enables a dyeless visualization of blood flow in different retinal layers. This noninvasive technology is finding increasing acceptance in clinical practice and a growing field of clinical research is focusing on the microvascular changes evaluated using OCT-A in different retinal diseases [2,4]. The FAZ area has been evaluated using OCT-A in healthy subjects [11], in patients with systemic diseases [3,8,14] and in subjects with different retinal diseases including retinal vein occlusion, diabetic retinopathy, retinitis pigmentosa, sickle cell disease and Stargardt disease, after macular hole surgery and in patients with Behçet uveitis [6,7, 15–19].

In current clinical practice, measurements of the FAZ area are facilitated through the use of many different devices. However, the repeatability of measurements provided by some devices and their interchangeability with those provided by other instruments has not yet been evaluated. We evaluated the agreement between the Spectralis OCT-A (Heidelberg), Canon OCT-HS100 (Canon) and RTVue XR Avanti (Optovue) and also assessed the intra-device repeatability and reproducibility with regard to the area of the FAZ. Repeatability and reproducibility of the three devices was excellent. This is in line with previous studies in the literature [20–23]. However, measurements of the FAZ using the Canon and Heidelberg device have not been thoroughly evaluated before in the literature. Previous studies either did not evaluate the agreement between the different devices or the reproducibility [9]. Moreover, FAZ measurement was often performed using ImageJ software [10]. This approach would probably not be applicable in the clinical practice.

In our study measurements of the FAZ area showed an excellent repeatability and reproducibility in healthy eyes in all three devices and there was no significant difference in ICC between the three devices. However, the high level of reproducibility in our study was achieved through the participation of experienced trained readers and an expert examiner. It is unclear, whether similar results are achievable with less intensively trained individuals. For instance, Pilotto et al. showed a lower intraoperator and interoperator agreement at the deep capillary plexus using the Spectralis device [23]. In their study, more than one eye of one subject was included, which might have had an impact on the results. Moreover, an OCT-A scan pattern of 15*5° was used compared to a 10°x10° OCT-A scan in our study.

The agreement analysis in our study confirmed statistically significant differences between measurements obtained using the three different devices. The Heidelberg device measured a significantly larger FAZ area compared to the Optovue and the Canon device. The FAZ area measured using the Canon device was also slightly, but significantly smaller than the FAZ area measured using the Optovue device. Nevertheless, there was a strong correlation between measurements obtained with the three different devices (Fig 2), and the degree of agreement might be acceptable for daily clinical use. However, for clinical research measurements of the FAZ using these three different devices are not directly interchangeable.

In this study, repeated measurements were performed on one eye of each healthy subject with the three different devices, thus improving the validity of statistical analysis. However, this study has important limitations. First, we did not include patients with retinal pathologies or postoperative conditions and the sample size was relatively small. Further studies in patients with different retinal pathologies are therefore needed. Moreover, at the time of the measurements, none of the devices used a projection-resolved (PR)-OCT-A algorithm, increasing the probability of incorrect visualization of the vasculature in the deeper layers. The impact of (PR)-OCT-A algorithm has to be evaluated in further studies.

In conclusion, The FAZ area measurements in healthy subjects showed good repeatability and reproducibility with the three different devices, and the ICC did not differ significantly between any pairs of the three devices. The agreement analysis in this study demonstrates slight, but significant differences in the measurement of the FAZ between the three devices. This should be taken into consideration in patient follow-up as well as in clinical research.

## Supporting information

S1 Table Mean and standard deviation of foveal avascular zone area (mm^2^) and signal strength index (SSI) or quality index (QI), respectively, of three measurements (m1, m2, m3) and two readers (R1, R2) with the different devices. (DOCX)
