# Supplementary material for: Repeatability, reproducibility and agreement of foveal avascular zone measurements using three different optical coherence tomography angiography devices
Source: PLoS One. 2018 Oct 18;13(10):e0206045. doi: 10.1371/journal.pone.0206045 (PMC6193722; doi:10.1371/journal.pone.0206045)
Supplement: S1 Table — (DOCX) [file pone.0206045.s001.docx]

|  | **m1** | | | **m2** | | **m3** | | | **overall** | | | |  |
| --- | --- | --- | --- | --- | --- | --- | --- | --- | --- | --- | --- | --- | --- |
|  | SCP | DCP | | SCP | DCP | SCP | | DCP | SCP | DCP | | |  |
| **Optovue** |  |  | |  |  |  | |  |  |  | | |  |
| R1 | 0.309 ± 0.087 | 0.328 ± 0.092 | | 0.309 ± 0.090 | 0.328 ± 0.093 | 0.308 ± 0.089 | | 0.330 ± 0.088 | 0.308 ± 0.087 | 0.329 ± 0.090 | | |  |
| R2 | 0.314 ± 0.090 | 0.343 ± 0.103 | | 0.318 ± 0.094 | 0.350 ± 0.097 | 0.314 ± 0.092 | | 0.344 ± 0.093 | 0.315 ± 0.091 | 0.346 ± 0.097 | | |  |
| SSI | 73.333 ± 8.149 | | | 73.583 ± 7.655 | | 70.542 ± 8.119 | | | 72.486 ± 7.986 | | | |  |
| **Canon** |  |  | |  |  |  | |  |  |  | | |  |
| R1 | 0.295 ± 0.093 | 0.294 ± 0.092 | | 0.295 ± 0.088 | 0.297 ± 0.085 | 0.296 ± 0.089 | | 0.304 ± 0.090 | 0.295 ± 0.089 | 0.298 ± 0.088 | | |  |
| R2 | 0.301 ± 0.101 | 0.298 ± 0.106 | | 0.300 ± 0.086 | 0.303 ± 0.100 | 0.298 ± 0.091 | | 0.313 ± 0.105 | 0.300 ± 0.092 | 0.305 ± 0.103 | | |  |
| SSI | 7.583 ± 1.349 | | | 7.417 ± 1.472 | | 7.542 ± 1.179 | | | 7.514 ± 1.280 | | | |  |
| **Heidelberg** |  |  | |  |  |  | |  |  |  | | |  |
| R1 | 0.326 ± 0.089 | 0.331 ± 0.089 | | 0.328 ± 0.090 | 0.333 ± 0.093 | 0.332 ± 0.097 | | 0.349 ± 0.099 | 0.329 ± 0.091 | 0.338 ± 0.093 | | |  |
| R2 | 0.329 ± 0.100 | 0.324 ± 0.097 | | 0.326 ± 0.093 | 0.326 ± 0.093 | 0.332 ± 0.104 | | 0.345 ± 0.099 | 0.329 ± 0.098 | 0.332 ± 0.095 | | |  |
| QI | 39.278 ± 3.663 | | | 39.875 ± 3.379 | | 39.292 ± 3.928 | | | 39.278 ± 3.663 | | | |  |
|  |  | |  |  | | |  |  | | |  |  |  |

**S1 Table.** Mean and standard deviation of foveal avascular zone area (mm²) and signal strength index (SSI) or quality index (QI), respectively, of three measurements (m1, m2, m3) and two readers (R1, R2) with the different devices.
